# Dual‐Loaded Nanocarriers With High Stability in Gastrointestinal Tract for Type 2 Diabetes and Hypertension Prevention

**DOI:** 10.1002/fsn3.70394

**Published:** 2025-06-08

**Authors:** Melike Yücetepe, Mehmet Şükrü Karakuş, Merve Akalan, Kamile Bayrak Akay, Hidayet Sağlam, Asliye Karaaslan, Bülent Başyiğit, Mehmet Karaaslan

**Affiliations:** ^1^ Food Engineering Department, Engineering Faculty Harran University Şanlıurfa Türkiye; ^2^ Harran University, Application and Research Center for Science and Technology Şanlıurfa Türkiye; ^3^ Molecular Biology and Genetics Department, Arts and Sciences Faculty Kilis 7 Aralık University Kilis Türkiye; ^4^ Harran University, Vocational School, Food Processing Programme Şanlıurfa Türkiye

**Keywords:** bioaccessibility, hypertension, nanoliposomes, phenolics, pomegranate seed‐derived peptides, type 2 diabetes

## Abstract

Today, synthetic drugs with side effects on health are used as agents for treating diseases. Moreover, these agents are utilized in the treatment of one specific disease. Consequently, it is essential to develop natural systems that do not harm health and instead foster healing for a range of diseases upon consumption. Therefore, the study focused on examining the behavior of nanocarriers containing the combination of *Quercus infectoria* gall‐derived phenolic powder (GP) and pomegranate seed‐derived peptide (BP) on type 2 diabetes and hypertension in the in vitro gastrointestinal tract. Firstly, the plausible conditions (enzyme type: Alcalase and enzyme‐to‐protein ratio: 1/20, w/w) for BP production were performed. Nanoliposomal systems having four different natures were called B‐NL (nanoliposome prepared without phyto‐active), GP‐NL (phenolic‐loaded nanoliposome), BP‐NL (peptide‐loaded nanoliposome), and GPBP‐NL (both phenolic and peptide‐loaded nanoliposome). The conversion of protein to peptide resulted in the disappearance of characteristic protein bands in SDS‐PAGE. FTIR spectra indicated that physical interactions predominated within nanocarriers. SEM images showed the dominance of spherical structures for all samples. Particle size (228.90‐364.30 nm), polydispersity index (PDI: 0.10‐0.48), zeta potential (14.50‐17.00 mV), and encapsulation efficiency (EE: 86.33%–90.30%) of nanoliposomes were elaborated. Bioaccessibility of GP (40.05%) and BP (38.09%) was lower than those of their nano‐encased forms (85.92%–87.03%). Also, GP‐NL, BP‐NL, and GPBP‐NL displayed superior inhibition activity toward key enzymes associated with type 2 diabetes and hypertension in the micellar phase compared to their uncoated forms. Ultimately, innovative bifunctional nanocarriers with high potential for treating diabetes and hypertension were developed.

## Graphical abstract

Multifunctional nanoliposomes containing phenolics and peptides were designed. Nanoparticles exhibited desirable antidiabetic actions in the micellar phase. Hypertension‐limiting systems were produced for evaluating in vivo applications.

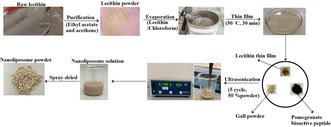

## 1 Introduction

In recent decades, ensuring the physiological well‐being and health of individuals has emerged as a core objective in various research. De novo lifestyles introduce elevated standards that focus on physical activity, stress management, and nutritious eating habits (Brazil et al. 2021). Consuming low‐fat, whole‐grain, and plant‐based foods is a key element of a well‐planned diet conferring health benefits (Dutton 2023). A strong tendency toward the consumption of bioactive compound‐rich foods was apparent lately. The functional food sector has bloomed to produce diet‐specific, micronutrient‐rich, health‐promoting foodstuffs unlike anything preceding them (Alongi and Anese 2021). This food research continues even in the field of oral and dental health (Abedinia et al. 2025). In addition, sustainable microbial‐supported strategies for applications such as food processing produced from biomaterials for health purposes have begun to be investigated (İncili et al. 2025). Epidemiologic studies accentuated the therapeutic action of bioactive compounds on biological systems and relate to reduced risk of cardiovascular diseases, diabetes, and tumor formation (Sindhu et al. 2021). Such wise compounds defend complex genetic polymers, polypeptide chains, and cellular organelles against the detrimental effects of reactive oxygen species (Akbarian et al. 2022; Unuofin and Lebelo 2020).

Of these compounds, bioactive peptides (BPs) and phenolic compounds particularly exert ameliorative effects on living organisms. Obesity, hypertension, and hyperglycemia are associated with oxidative stress and impair metabolic activities of pancreatic α‐amylase, intestinal α‐glucosidase, and angiotensin‐I‐converting enzymes (ACE) (Irondi et al. 2019; Jia et al. 2021). Hyperglycemia, causing wounds related to diabetes (Zeng et al. 2025) and hypertension (Zhao et al. 2024), may trigger and exacerbate cardiovascular defects. Exterminating such disorders became a key challenge to boost the quality of life (Glovaci et al. 2019; Khumaedi et al. 2019). Additionally, type 2 diabetes has been associated with neurological disorders (Wei et al. 2024). Synthetic drugs prevailed in the clinical treatments aiming to inhibit the diabetic (Chen et al. 2025) and hypertensive enzyme activities (Fagherazzi and Ravaud 2019). The respective drugs are administered to treat specific diseases. For example, SGLT2 inhibitors may help improve kidney and heart health and assist people with type 2 diabetes in managing their blood sugar (Shen et al. 2023). Possible side effects of synthetic medications may include, but are not limited to, hypersensitivity, gastrointestinal complaints, dizziness, and nausea. Clearly, scientific literature and the industry are striving to incorporate alternative sustainable natural materials into the relevant applications. Plant‐derived BPs (Xue et al. 2021) and phenolics (Cam et al. 2020) are viewed as excellent alternatives to synthetic enzyme suppressors due to their minimal side effects and strong enzyme inhibitory activity. BPs have become increasingly popular in today's functional foods due to their ability to improve bodily functions and lower the risk of diseases (Zhu, Ma, et al. 2024). Multifunctional BPs exert antihypertensive (Li et al. 2022), antidiabetic (Elam et al. 2021), antimicrobial (Tang et al. 2018), osteogenic (Zhu, Cheng, and Du 2024; Zhu, Ma, et al. 2024), antioxidant (Wong et al. 2020), and anti‐inflammatory (Liu et al. 2025) activities with low allergenicity, toxicity, and hypocholesterolemic properties (Udenigwe and Aluko 2012). BPs are vulnerable to enzymes in the gastrointestinal tract, leading to their degradation and loss of effectiveness (Liu et al. 2023). Furthermore, when incorporated into foods, they may exhibit inherent instability and interact with the components of the food. Given these challenges, it is essential to enhance the resilience and stability of BPs (Mor et al. 2021). As for phenolics, they are a category of natural compounds recognized for their strong antihypertensive, antidiabetic, and antioxidant properties (Cam et al. 2020; Cheng et al. 2024). Despite their potential, the use of bioactive compounds in food and pharmaceutical applications is restricted by factors such as sensitivity to light, temperature, and pH; issues with solubility, low stability during processing and storage, susceptibility to oxidation, and interactions with other substances (Ashraf et al. 2025). The combination of BP and phenolics results in the creation of robust complexes that demonstrate improved functionality (Ceylan et al. 2022). Initial studies indicate that complexes facilitate the transport of BP/phenolics through the digestive tract with minimal damage, while simultaneously enhancing their bioaccessibility and antioxidant activity (Garcia‐Mora et al. 2015; Lin et al. 2023). On the other hand, most of the bioactive compounds display low solubility and inadequate penetrability to the inner space of cellular compartments. Therefore, encapsulation of multiple compounds within the versatile carrier systems is imperative to increase their stability and bioaccessibility (Liu et al. 2023; Rafiee et al. 2017; Esmaeili et al. 2024). In the encapsulation of bioactive substances, proteins, polysaccharides, and lipids are utilized as carrier systems. Proteins, particularly when carrying BPs, could interact with them because of their diverse structure and limited mobility. Polysaccharides are cost‐effective; however, they possess the ability to interact with peptides, potentially resulting in the loss of their bioactivity (Fathi et al. 2014). In contrast, lipid‐based carriers, such as nanoliposomes (NLs), have gained prominence due to their elevated safety profiles and lack of immunological reactivity. NL is a modern nanosized delivery tool made of phospholipids to preserve and transmit the functional compounds within the gastrointestinal tract (Chotphruethipong et al. 2020). Nanoliposome vesicles enclose a hydrophobic tail and a hydrophilic cap and contain enough room to accommodate polar and nonpolar moieties (McClements 2015). Such colloidal systems could further be employed to improve surface area, solubility, and bioavailability of natural components while protecting biologically active substances against negative external factors. Encapsulation of bioactive components within bimolecular compartments of such systems is considered a favorable approach ascribed to matching advantages of nanoliposomes (Chotphruethipong et al. 2020). To make it more concrete, NLs protect bioactive compounds in the gastrointestinal tract from pH fluctuations, interactions with metal ions, enzymes, and free radicals, ensuring their delivery to the relevant region (Amiri et al. 2023). NL systems sheltering collagen peptide from sturgeon fish (Kung et al. 2025), osteogenic peptides (Zhu, Cheng, and Du 2024; Zhu, Ma, et al. 2024), oleaster‐seed protein peptides (Ahaninjan et al. 2025), shrimp waste‐derived bioactive peptides (Khalatbari et al. 2024), fenugreek seed extract (Ashraf et al. 2025), *Salvia leriifolia* Benth phenolics (Nasrabadi et al. 2024), and phenolics from pistachio hulls (Oskoueian et al. 2020) were fabricated recently. As shown in prior studies, BPs and phenolics were individually integrated into nanoliposome systems and subsequently characterized. To the best of our knowledge, there are currently no bifunctional NL matrices that concurrently incorporate both bioactive components, namely BP and phenolics, documented in the existing literature. For this purpose, firstly, BP from pomegranate seeds and phenolic powders (GPs) from *Quercus infectoria* galls were obtained. In the second stage, the objective was to produce and characterize bifunctional nanoliposome matrices that incorporate two bioactive components simultaneously, rather than focusing solely on nanoliposomes that contain only one bioactive component. The degree of hydrolysis, antioxidant activity, amino acid composition, and sodium dodecyl sulfate polyacrylamide gel electrophoresis (SDS‐PAGE) pattern of BPs were characterized. NL matrices containing BP, GP, and the BP/GP composites were produced by the thin film hydration technique. They were characterized via FTIR spectroscopy, SEM, particle size, polydispersity index (PDI), zeta potential, and encapsulation efficiency tests. Also, the bioaccessibility, antidiabetic, and ACE inhibitory activities of BP, GP, and the BP/GP complex within NLs in the in vitro gastrointestinal tract were evaluated. Ultimately, in the current study, NL matrices, multifunctional vesicles with high antidiabetic and antihypertensive activities in the in vitro gastrointestinal system, were designed.

## 2 Materials and Methods

### 2.1 Materials

Gall ( *Quercus infectoria* ) was purveyed by a manufacturer in Gaziantep, Türkiye. This plant was collected between August and October in the mountainous and rural areas of Gaziantep province. Pomegranate seeds (PSs) were supplied from Mavideniz Food Co. in Isparta, Türkiye. Chloroform (67‐66‐3), formaldehyde (50‐00‐0), NaOH (sodium hydroxide) (1310‐73‐2), TCA (trichloroacetic acid) (76‐03‐9), sodium tetraborate (1330‐43‐4), SDS (sodium dodecyl sulfate) (151‐21‐3), OPA (o‐Phthalaldehyde) (643‐79‐8), methanol (67‐56‐1), β‐mercaptoethanol (60‐24‐2), DPPH (2,2‐Diphenyl‐1‐picrylhydrazyl) (1898‐66‐4), ABTS (2,2′‐Azino‐bis(3‐ethylbenzothiazoline‐6‐sulfonic acid) diammonium salt) (30931‐67‐0), trolox (53188‐07‐1), potassium persulfate (7727‐21‐1), 2,4,6‐tris(2‐pyridyl)‐s‐triazine (3682‐35‐7), iron (II) chloride (7758‐94‐3), ammonium acetate (631‐61‐8), ethanol (64‐17‐5), neocuproine (484‐11‐7), copper (II) chloride (7447‐39‐4), formic acid (64‐18‐6), acetonitrile (75‐05‐8), trizma base (77‐86‐1), NaCl (sodium chloride) (7647‐14‐5), glycerol (56‐81‐5), bromophenol blue (115‐39‐9), coomassie brilliant blue G‐250 (6104‐58‐1), folin‐ciocalteau (F9252), sodium carbonate (497‐19‐8), gallic acid (149‐91‐7), bradford reagent (B6916), calcium chloride (10043‐52‐4), HCl (hydrochloric acid) (7647‐01‐0), bile (8008‐63‐7), 4‐nitrophenyl‐α‐d‐glucopyranose (3767‐28‐0), starch (9005‐84‐9), sodium potassium tartrate (6381‐59‐5), 3,5‐dinitrosalicylic acid (609‐99‐4), Hepes (7365‐45‐9), HHL (N‐hippuryl‐His‐Leu hydrate) (207386‐83‐2), hippuric acid (495‐69‐2), and ethyl acetate (141‐78‐6) were purchased from Merck (Darmstadt, Germany). Analytical purity of solvents and reagents was utilized unless stated otherwise. Enzymes, Vegazym HC and Fructozym P6‐XL, were purveyed from Erbslöh (Geisenheim, Germany); pepsin and Alcalase were provided by Novozymes Co. (Kalundborg, Denmark); α‐glucosidase, α‐amylase, lipase, trypsin, and ACE were supplied by Sigma Aldrich Co. (St. Louis, MO, USA).

### 2.2 Fabrication of Raw Materials and Nanoliposome Systems

#### 2.2.1 Gall Phenolic Powder

Gall phenolics were extracted by microwave‐assisted (Sineo, Mass II Plus, Shanghai, China) enzymatic extraction method. The enzyme amount/type and microwave conditions were optimized (all datasets regarding optimization have not yet been published). Firstly, raw material was dried (moisture < 10% ± 1%) and the inner part was separated. Ten grams of ground gall (200–250 μm) was mixed with 90 mL of distilled water. After the pH of the solutions was set to 4.0 ± 0.1, the final volume was fulfilled to 100 mL. Enzyme additions were performed according to the experimental design. The extraction was conducted for 2 h at 50.0°C ± 1.5°C in a water bath (Nüve ST 402, Ankara, Turkey). Enzyme inactivation was performed at 90.0°C ± 2.0°C for 10 min. After centrifugation (1420 *g*, 10 min) (NF 1200R, Nüve, Ankara, Turkey), the total phenolic content (TPC) was detected in the supernatant. The appropriate enzyme type/ratio (0.020 mL/L Vegazym HC and 0.015 mL/L Fructozym P6‐XL) was evaluated according to the maximum TPC.

For the microwave‐assisted enzymatic extraction, solutions containing gall and distilled water at a ratio of 1:10 (w:v) were exposed to enzymatic applications (0.020 mL/L Vegazym HC and 0.015 mL/L Fructozym P6‐XL), and the final mixtures were placed in the relevant part of the microwave. Experiments were implemented at various microwave powers (250–500 W) and processing times (10–30 min). Optimal conditions with maximum TPC were 463.24 W and 26.21 min. Extracts obtained under these conditions were fed to a spray‐dryer with an inlet temperature of 140.0°C ± 3.0°C and a feed flow rate of 8 ± 1 mL/min (B15, Unopex, Izmir, Turkey). The gall phenolic powders (GPs) were kept at 4.0°C ± 0.5°C until analyses.

#### 2.2.2 Pomegranate Seed Protein

Microwave‐assisted enzymatic extraction conditions of pomegranate seed proteins (PPs) were optimized (all datasets regarding optimization have not yet been published). In the enzymatic extraction process, distilled water (90 mL) was added to 10 g of de‐fatted pomegranate seeds. After adjusting the pH of the solution to 4.0 ± 0.1, the final volume was completed to 100 mL with distilled water. Enzyme(s) were added at the relevant ratios to this mixture and incubated in a water bath (50.0°C ± 1.5°C) for 90 min. Next, the pH value was set to 9.5 ± 0.1 and kept at 50.0°C ± 1.5°C for 30 min. Mixtures were centrifuged (1420 *g*, 10 min). The protein content was defined in the supernatant. The appropriate enzyme type/ratio (0.030 mL/L Vegazym HC and 0.010 mL/L Fructozym P6‐XL) was evaluated according to the maximum protein content.

For the microwave‐assisted enzymatic extraction, the pH of the mixtures containing de‐fatted pomegranate seed and distilled water (1:10, w:v) was adjusted to 4.0 ± 0.1, and the enzymes (0.030 mL/L Vegazym HC and 0.010 mL/L Fructozym P6‐XL) were added. The extraction process was carried out at different conditions (microwave power: 250–500 W and processing time: 10–60 min) according to the experimental design. Then, pH was arranged to 9.5 ± 0.1, and solutions were held at 50.0°C ± 1.5°C for 30 min. The amount of protein in the centrifuged supernatant was calculated. Supernatant with the maximum protein content was obtained at 463.39 W for 52.68 min and dried using a spray‐dryer (140.0°C ± 3.0°C, 8 ± 1 mL/min flow rate). Powders were utilized to produce bioactive peptides (BPs).

#### 2.2.3 Bioactive Peptides

The method of Evangelho et al. (2017) was applied with minor modifications to produce bioactive peptides (BPs) (Evangelho et al. 2017). The enzyme type/ratio (pepsin and/or Alcalase, 0–1 g enzyme/20 g protein) was optimized to maximize the degree of hydrolysis. Briefly, the PPs were dissolved in PBS (phosphate buffer saline: 10 mM, pH 7.0 ± 0.1) (1:10, w:v), and these solutions were treated with pepsin (37.0°C ± 1.0°C and pH 2.0 ± 0.1) for 3 h and/or Alcalase (50.0°C ± 1.5°C and pH 8.0 ± 0.1) for 3 h according to the 5 trials as given in Table 1. At the end of the process, enzyme inactivation was performed (90.0°C ± 2.0°C, 10 min). Next, hydrolysates were passed through centrifugal‐type ultrafiltration tubes (Amicron Ultra‐15 Centrifugal Filters) with 10 kDa molecular weight. The collected filtrates or their freeze‐dried (FD4E, CoolerMed, İstanbul, Türkiye) form were used for further analyses.

**TABLE 1:** Experimental design for bioactive peptide production^a^, hydrolyzation degree and antioxidant activity.

Trials	Pepsin	Alcalase	Titration tech. (%)	TCA‐Bradford tech. (%)	OPA reagent tech. (%)	DPPH (mmol TEAC/g sample)	ABTS (mmol TEAC/g)	FRAP (mmol TEAC/g)	CUPRAC (mmol TEAC/g)
1	1 g	—	5.63 ± 0.05^e^	6.18 ± 0.57^d^	1.25 ± 0.01^c^	15.23 ± 1.21^b^	22.77 ± 0.83^d^	0.36 ± 0.02^c^	0.97 ± 0.08^c^
2	0.75 g	0.25 g	6.97 ± 0.19^d^	7.72 ± 0.25^cd^	1.60 ± 0.02^c^	16.89 ± 0.71^a^	37.26 ± 1.02^c^	0.59 ± 0.01^b^	1.85 ± 0.10^b^
3	0.50 g	0.50 g	7.72 ± 0.09^c^	9.36 ± 0.63^c^	1.71 ± 0.01^bc^	18.04 ± 0.27^a^	51.07 ± 1.52^b^	0.60 ± 0.03^b^	1.92 ± 0.05^b^
4	0.25 g	0.75 g	8.45 ± 0.24^b^	10.86 ± 0.67^b^	2.14 ± 0.01^b^	18.38 ± 0.16^a^	54.73 ± 1.15^b^	0.65 ± 0.02^b^	2.08 ± 0.07^b^
5	—	1 g	9.92 ± 0.05^a^	16.83 ± 0.89^a^	3.35 ± 0.28^a^	19.31 ± 0.24^a^	65.88 ± 0.74^a^	0.83 ± 0.02^a^	2.79 ± 0.00^a^

*Note:* Results are given as mean ± standard deviation (*n* = 3). Different lowercase letters in the same column indicate the difference between experiments (*p* < 0.05).

^a^ Enzymes are given according to the enzyme/substrate (protein) ratio of 1:20 (w/w).

#### 2.2.4 Nanoliposome Systems

**FIGURE 1:**
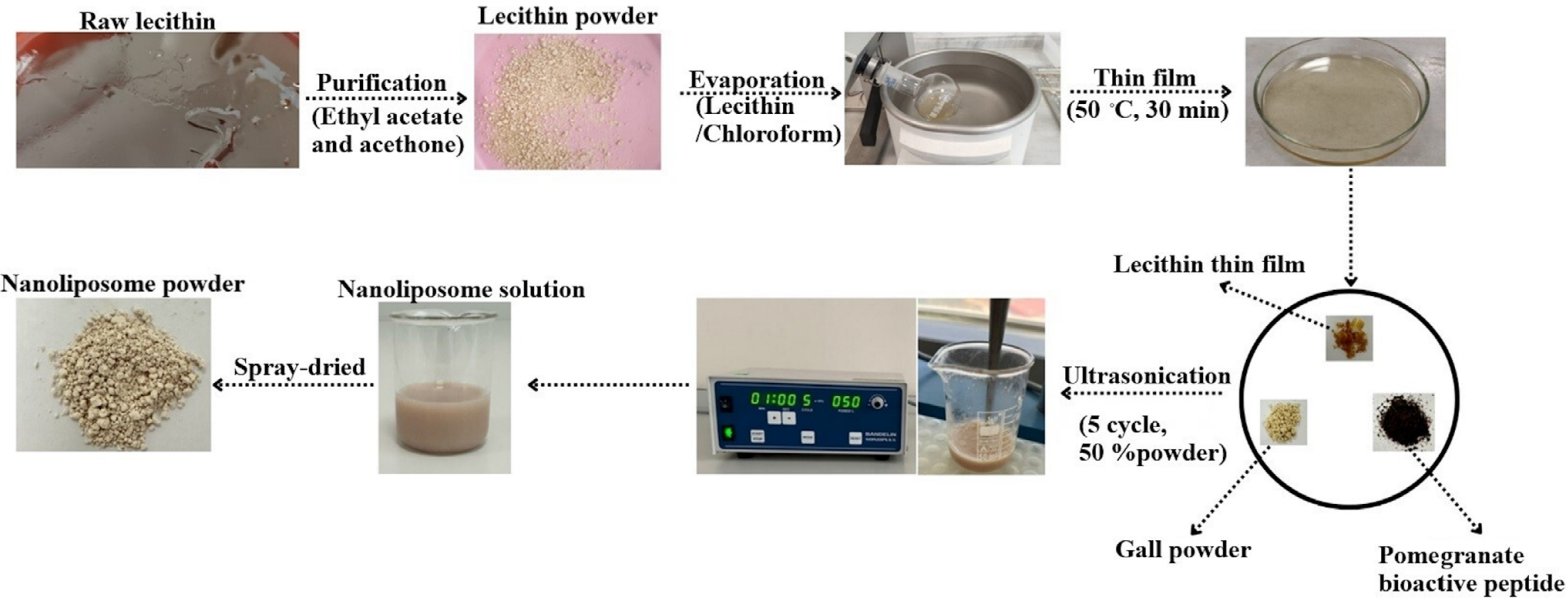
Flowchart for nanoliposome fabrication.

A previous method for the construction of nanoliposomes using the thin‐layer film hydration technique was performed with some modifications (Figure 1) (Mosquera et al. 2014). B‐NL (nanoliposome prepared without phyto‐active compounds), GP‐NL (phenolic‐loaded nanoliposome), BP‐NL (peptide‐loaded nanoliposome), and GPBP‐NL (both phenolic‐ and peptide‐loaded nanoliposome) were fabricated. For this, the purified lecithin was dissolved in chloroform. The organic phase was eliminated by a rotary evaporator (R‐210, Buchi, Flawil, Switzerland). The remaining part was transferred to the petri dish and kept at 50.0°C ± 1.5°C for 30 min to obtain film layers. To evolve the nanoliposome (NL) system, the ratio of thin films to GP or/and BP was adjusted to 1:4 (w/w) throughout the experiments. All NL solutions prepared in 50 mL PBS (10 mM, pH 7.0 ± 0.1) were held in a water bath (60.0°C ± 1.5°C, 30 min) and exposed to an ultrasonicator (Sonopuls UW2070, Bandelin, Berlin, Germany) at 1 min intervals (5 cycles and 50% power). Sonicated solutions were cooled down and used for analyses (freshly prepared NLs were used for all experiments).

### 2.3 Analyses

#### 2.3.1 Degree of Hydrolysis

The degree of hydrolysis (DH) of the BPs was detected in three diverse methods, including titration, TCA, and OPA techniques.

For the titration technique, 1.5 g freeze‐dried BP was dissolved in 50 mL distilled water, and pH was set to 7.0 ± 0.1 (0.1 N NaOH). Next, 10 mL of formaldehyde (38%, v/v) was added to this solution and was held at room temperature (5 min). The titration was done with NaOH (0.1 N) until the pH reached 8.5 ± 0.1. The amount of NaOH used was noted, and the DH was determined by the following equations (Equations 1 and 2) (Noman et al. 2018).

(1) $$\text{Free amino group}\;\left(\%\right)=\frac{A\times B\times 0.014007}{C}\times 100$$

(2) $$\mathrm{DH}\;\left(\%\right)=\frac{\text{free amino group}\;\left(\%\right)}{\text{total nitrogen}\;\left(\%\right)}\times 100$$

where, A: amount of NaOH (mL), B: concentration of NaOH (0.1 M), and C: amount of sample (1.5 g).

For the TCA technique, the mixture of 1 mL filtrate and 1 mL TCA (0.44 M) was centrifuged (8000 *g*, 4.0°C ± 0.5°C, 10 min). Soluble protein in the supernatant was measured by the Bradford method (Mohammadi et al. 2022). The DH was found out with the following equation (Equation 3).

(3) $$\mathrm{DH}\;\left(\%\right)=\frac{\text{the amount of soluble protein in supernatant}}{\text{the amount of protein in the sample}}\times 100$$

For the OPA technique, 3.4 mL OPA reagent containing 25 mL 100 mM sodium tetraborate solution, 2.5 mL 20% (w/v) SDS, 40 mg OPA (in 1 mL methanol), and 21.5 mL distilled water was stirred with 10 μL filtrate. Then, 100 μL β‐mercaptoethanol was added to this mixture and held at 35.0°C ± 1.0°C (2 min). At the end of the period, the absorbance at 340 nm was read. The DH was defined according to the equation (Equation 4) (Morais et al. 2013).

(4) $$\mathrm{DH}\;\left(\%\right)=\frac{A\times 1.934\times \mathrm{DF}}{C}$$

where, *A*: absorbance of the samples, DF: dilution factor, and *C*: concentration of the protein sample (g/L).

#### 2.3.2 Antioxidant Activity

The antioxidant activities of BP were set on with 4 different methods (DPPH radical scavenging activity, ABTS, FRAP, and CUPRAC). The results were reported as mmol Trolox‐equivalent (TEAC) per gram of the sample.

For the DPPH radical scavenging activity method, 3.9 mL of DPPH solution (25 mg/L) and 0.1 mL samples were stirred and kept in the dark (30 min). The absorbance was read (515 nm) using a UV–VIS spectrophotometer (UV‐1700, Shimadzu, Kyoto, Japan) (Çam et al. 2009).

For the ABTS assay, a 7.5 mM ABTS radical solution was prepared and combined with a 2.45 mM potassium persulfate solution, followed by incubation in the dark for 16 h. Then, this solution was diluted with PBS (pH 7.6 ± 0.1) until the 0.700 ± 0.02 absorbance value at 734 nm. Two mL of ABTS solution was mixed with samples at different concentrations (20, 40, 60, and 80 μL), and the absorbances were recorded after 6 min (Çam et al. 2009).

For the FRAP method, this analysis was performed according to the method of Alsataf et al. (2021). The samples (150 μL) were treated with 2850 μL FRAP reagent (25 mL 30 mM acetate solution, 2.5 mL 10 mM 2,4,6‐tris(2‐pyridyl)‐s‐triazine, and 2.5 mL 20 mM iron (II) chloride). Samples were incubated for 30 min, and absorbances were noted at 593 nm (Alsataf et al. 2021).

For the CUPRAC assay, samples (0.1 mL) were placed in a glass tube including 1.0 M ammonium acetate buffer solution (1 mL), 7.5 × 10^−3^ M ethanolic neocuproine solution (1 mL), and 0.01 M copper (II) chloride solution (1 mL). The last volumes were made up to 4.1 mL with distilled water. After 30 min, absorbances were read at 450 nm (Alsataf et al. 2021).

#### 2.3.3 Amino Acid Composition

The amino acid composition of PP and BP was detected by the LCMS‐8040 device (Shimadzu, Kyoto, Japan). A mixture of solvents A (formic acid: 0.05% (v/v)) and B (acetonitrile) was prepared as a mobile phase (A:B, 30:70, v/v). Agilent C18 XDB column (3.5 μm 3 × 150 mm), temperature (30.0), flow rate (0.07 μL/min), injection volume (40 μL), heat (500.0°C), nebulizing gas (3 L/min), and drying gas (20 L/min) were constant during analysis (Saglik et al. 2019).

#### 2.3.4 SDS PAGE

SDS PAGE was realized according to a previous method (12% (w/v) separating gel and 5% (w/v) stacking gel) (Amiri et al. 2024). PP and BP (10 mg/mL) were dissolved in a buffer solution (0.1 M Tris–HCl, 0.1 M NaCl, and 10% (w/v) SDS) (Ata et al. 2022). Samples were denatured at 90°C ± 2.0°C for 5 min (Abedinia et al. 2017). The solutions were blended with sample loading buffer containing Tris–HCl (5% (v/v), pH 6.8 ± 0.1), glycerol (4%, v/v), SDS (0.8%, w/v), bromophenol blue (0.02%, w/v), and β‐mercaptoethanol (2%, v/v) at a rate of 1:1 (v/v) and then kept at 95.0°C ± 2.0°C (10 min). A protein standard (11–190 kDa) was used as a molecular weight marker. After electrophoresis, Coomassie Brilliant Blue G‐250 was performed to color the gel.

#### 2.3.5 Fourier Transform Infrared Spectroscopy

Fourier transform infrared spectroscopy (FTIR) spectroscopy (IRTracer‐100, Shimadzu, Kyoto, Japan) was utilized to define the functional groups of lecithin, GP, BP, and NLs. The analysis was performed with an intention of 1 cm^−1^ and between 4000 and 400 cm^−1^ at ambient temperature (Aisha et al. 2014).

#### 2.3.6 Morphology of Nanoliposomes

The surface morphological structures of spray‐dried nanoliposome powders were monitored with scanning electron microscopy (SEM) (ZEISS Sigma 300 Field Emission SEM, Oberkochen, Germany) (Kasapoğlu et al. 2024).

#### 2.3.7 Particle Size, PDI, and Zeta Potential

The particle size, PDI, and zeta potential (the surface electrical charge) of the NLs were defined with a dynamic light scattering system (Nano ZS90, Malvern Instruments, Worcester, UK) at room temperature (Sarabandi, Jafari, et al. 2019; Sarabandi, Mahoonak, et al. 2019).

#### 2.3.8 Encapsulation Efficiency

Encapsulation efficiency (EE) was defined considering previous research with some modifications (Gorzin et al. 2024). In this context, 2 mL NLs were centrifuged (2500 *g*, 10 min) using an Amicon Ultra‐15 centrifugal filter. The supernatant was collected to define the amount of non‐encapsulated BP or GP.

For TPC, diluted supernatant (0.4 mL) was stirred with 2 mL Folin‐Ciocalteau reagent (diluted 1:9, v/v) and 1.6 mL 7.5% (w/v) sodium carbonate. Solutions were incubated (60 min) in the dark place, and the absorbances were noted at 765 nm. The results were stated as mg gallic acid equivalent (mg GAE/g) per gram of sample (Singleton and Rossi 1965).

The protein content in the supernatant was defined according to the Bradford method (1976) (Bradford 1976).

The EEs for phenolics and peptides were calculated by the following equations (Equations 5 and 6).

(5) $$\mathrm{EE}\;\left(\%\right)=\frac{\text{initial phenolic content}-\text{nonencapsulated phenolic content}}{\;\text{initial phenolic content}}\times 100$$

(6) $$\mathrm{EE}\;\left(\%\right)=\frac{\text{initial protein content}-\text{nonencapsulated protein content}}{\;\text{initial protein content}}\times 100$$

#### 2.3.9 In Vitro Gastrointestinal Digestion

The gastrointestinal digestion procedure was conducted according to Minekus et al. (2014) with minor adjustments (Minekus et al. 2014).

For oral digestion, 5 mL NLs was homogenized with 3.5 mL simulated saliva fluid, and α‐amylase was added to the mixture at a concentration of 5 mg/mL. After 25 μL of CaCl_2_ (0.3 M) and 975 μL of distilled water were added, the pH was set to 7.0 ± 0.1. The solution was kept at 37.0°C ± 1.0°C for 2 min.

For gastric digestion, 3.75 mL simulated gastric fluid was homogenized with the oral bolus (5 mL). Pepsin (final concentration 250 U/mL) and 2.5 μL of 0.3 M CaCl_2_ were incorporated with this blend, and the pH was adjusted to 3.0 ± 0.1 with HCl (1 M). The distilled water (347 μL) was attached and incubated at 37.0°C ± 1.0°C for 2 h.

For intestinal digestion, 2.75 mL of the simulated intestinal fluid was added to 5.0 mL of chyme (gastric‐digested NLs). The related enzymes (trypsin: 100 U/mL, lipase; 2000 U/mL, α‐amylase; 10 U/mL) were suffixed to the mixture. Next, 50 mg of bile salt and 10 μL of CaCl_2_ (0.3 M) were annexed. The pH was set to 7.0 (1 M NaOH). After adding distilled water (328.50 μL), the incubation process was applied at 37.0°C ± 1.0°C for 2 h.

The bioaccessibility, antidiabetic, and ACE inhibition activity of the NLs containing bioactive compounds was accomplished directly in the final liquid (micellar) phase.

#### 2.3.10 Bioaccessibility

A previous method was used with some modifications to assign the bioaccessibility of nano‐encased bioactive components (Liu, Wang, et al. 2021). Three mL NLs containing GP, BP, or both bioactive compounds were centrifuged (1420 *g*, 15 min, 4.0°C ± 0.5°C). TPC (Singleton and Rossi 1965) and protein content (Bradford 1976) in the supernatant were defined with a UV–Vis spectrophotometer. The bioaccessibility was found out according to the following equation (Equation 7);

(7) $$\text{Bioaccessibility}\;\left(\%\right)=\frac{C_{d}}{C_{i}}\times 100$$

where, *C*_*d*_: the amount of bioactive compounds in the supernatant after digestion and *C*_*i*_: the amount of initial bioactive compounds in NLs.

#### 2.3.11 Antidiabetic Activity

The antidiabetic activities of NLs were established by evaluating their inhibitory behavior against α‐glucosidase and α‐amylase enzymes (McDougall et al. 2005).

For α‐glucosidase analysis, a glass tube containing a 50 μL sample, 1250 μL potassium phosphate solution (pH 6.8 ± 0.1), and 50 μL α‐glucosidase (0.5 units/mL) was placed in the water bath at 37.0°C ± 1.0°C. After incubation (5 min), 125 μL of 4‐nitrophenyl‐α‐d‐glucopyranose was added to initiate the reaction and held for 20 min. Then, 2 mL of sodium carbonate (0.1 M) was added to stop the reaction. The absorbance of the mixtures was measured at 400 nm.

For α‐amylase analysis, the glass tubes were kept in a water bath (37.0°C ± 1.0°C). Next, a 1 mL sample, 1 mL starch (1%, w/v), and 1 mL sodium phosphate buffer solution (20 mM, pH 6.9 ± 0.1) were added to these tubes. After 5 min, 1 mL α‐amylase (1 unit/mL) was added to initiate the reaction, and the solution was kept for 30 min. One mL of color reagent (2 M NaOH and 5.31 M sodium potassium tartrate prepared with 96 mM 3,5‐dinitrosalicylic acid) was supplemented to deactivate the reaction. Then, samples were boiled for 5 min, and absorbances were recorded at 540 nm. For the control and blank, no sample or enzyme was added to the tubes, respectively. The enzyme inhibitions were calculated according to the following formula (Equation 8).

(8) $$\text{Enzyme inhibition}\;\left(\%\right)=\frac{\left(\text{absorbance of control}-\text{absorbance of sample}\right)}{\;\text{absorbance of control}}\times 100$$

The results were expressed as IC_50_ values (mg/mL) (amount of sample required to inhibit 50% of the enzymes).

#### 2.3.12 Angiotensin‐I‐Converting Enzyme Inhibition Activity

The angiotensin‐I‐converting enzyme (ACE) inhibition activity of the NL was applied according to a prior study (Kittiphattanabawon et al. 2013). For this, a 50 μL sample (0.1%, w/v) was mixed with a buffer solution containing 300 mmol/L NaCl (pH 8.3 ± 0.1) and 50 mmol/L HEPES (N‐(2‐Hydroxyethyl)piperazine‐N′‐(2‐ethanesulfonic acid)‐HCl). ACE reagent (25 mL) prepared with the same solution was attached to this mixture and incubated (37.0°C ± 1.0°C, 5 min). The reaction was started with the addition of 50 mL of HHL (N‐hippuryl‐His‐Leu hydrate) solution (6 g/L). After 15 min, termination of the enzymatic reaction was done with 125 mL of HCl (1 M). The hippuric acid formed in the reaction was transferred to the ethyl acetate phase. After centrifugation (1200 *g*, 5 min), the organic phase (1 mL) in the supernatant was evaporated. The hippuric acid (residue) was dissolved in distilled water (1 mL), and the absorbances were determined at 228 nm. The sample blank and control blank underwent the same procedure, with the only difference being the addition of ACE solution into the reaction prior to the addition of HCl (1 M). ACE inhibition activity was calculated using the following equation (Equation 9).

(9) $$\mathrm{ACE}\;\text{inhibition activity}\;\left(\%\right)=\left(1-\frac{\left(\text{absorbance of sample}-a\text{bsorbance of sample blank}\right)}{\;\left(\text{absorbance of control}-a\text{bsorbance of control blank}\right)}\right)\times 100$$

The results were expressed as IC_50_ values (mg/mL) (amount of sample required to inhibit 50% of the enzymes).

#### 2.3.13 Statistical Analysis

All analyses were applied in triplicate. Means, standard deviations, and graphs were generated with OriginPro 2021b. The optimization process was performed by Design Expert 7.0 (Stat‐Ease Inc., Minneapolis, MN). Differences between means were defined with a one‐way ANOVA and Tukey's multiple comparison test at a 95% confidence level (*p* < 0.05). The data sets were created by the Statistical Package for the Social Sciences (SPSS) software (SPSS Inc., Chicago, IL, USA).

## 3 Results and Discussion

### 3.1 Optimization of Bioactive Peptides

Bioactive peptides could be catalyzed via various applications, namely enzymatic hydrolysis, microbial fermentation, chemical synthesis, chemical hydrolysis, digestion, cooking, ripening, and recombinant DNA technology (Cruz‐Casas et al. 2021). Among these applications, enzymatic hydrolysis is more favorable for the production of bioactive peptides because of its less troublesome processing steps, GRAS nature, regioselectivity, stereoselectivity, and so on (Chauhan and Kanwar 2020; He et al. 2019). There are issues that should not be overlooked in order to complete the process in a desirable manner, although this application exhibits these advantages. The selection of a suitable proteolytic enzyme type/ratio and process conditions (pH, temperature, *etc*.) is vital for an effective process (Evangelho et al. 2017). Here, in parallel with the abovementioned approaches, enzymatic hydrolysis in the fabrication of bioactive peptides from pomegranate proteins was exploited, and the reasonable conditions were examined by evaluating the impacts of enzyme types (pepsin and Alcalase)/ratios on DH (note: DH was detected by three different methods, including titration, TCA, and OPA reagent techniques) (Table 1) while the other factors were fixed by depending on enzyme specifications. The shifts in proteolytic enzyme type and proportions led to remarkable variations in the findings (*p* < 0.05). DH varied between 5.63% and 9.92% for the titration technique, 6.18% and 16.83% for the TCA technique, and 1.25% and 3.35% for the OPA reagent technique. The presence of Alcalase resulted in the promotion of these values. In other words, a positive correlation between DH and Alcalase concentration was obvious. That is, maximum values were detected in the hydrolysates catalyzed by Alcalase alone. Performing the process of protein hydrolysis using Alcalase rather than pepsin gives rise to better DH (Yan et al. 2023). Rice bran and soybean proteins treated by Alcalase instead of papain and certain kinds of enzyme mixtures display a tendency to give peptides with superior DH (Ahmadifard et al. 2016). In another study, advanced values in terms of DH were noted in casein hydrolysates exposed to Alcalase compared to neutrase‐ and trypsin‐produced counterparts (Yu et al. 2022). This superior behavior of Alcalase in the catalysis of peptides might be related to a series of explanations. Alcalase, a “serine endopeptidase”, possesses a wide variety of restriction sites. This characteristic feature provides a wide protease specificity to it. In other words, Alcalase could recognize a very broad range of amino acids (Phe, Trp, Tyr, Glu, Met, Leu, Ala, Ser, and Lys residues). Moreover, this enzyme has the ability to divide proteins in the center of the amino acid chain (Cui et al. 2022; Zhang et al. 2023). Regarding the relationship between DH and the biological activity of bioactive peptides, the higher the DH in the relevant product, the greater the antioxidant activity (Luo et al. 2014). These previous findings were supported by the antioxidant activity values of peptides in the present study. In other words, the outcomes of the antioxidant findings varied, influenced by the characteristics of the peptide content produced by various enzymes. Superior results were detected in Alcalase‐produced peptides (DPPH radical scavenging activity: 19.31 mmol TEAC/g, ABTS: 65.88 mmol TEAC/g, FRAP: 0.83 mmol TEAC/g, CUPRAC: 2.79 mmol TEAC/g). These values were 18.38 (DPPH radical scavenging activity), 54.73 (ABTS), 0.65 (FRAP), and 2.08 (CUPRAC) mmol TEAC/g for pepsin (0.25 g)/Alcalase (0.75 g)‐catalyzed ones; 18.04 (DPPH radical scavenging activity), 51.07 (ABTS), 0.60 (FRAP), and 1.92 (CUPRAC) mmol TEAC/g for pepsin (0.50 g)/Alcalase (0.50 g)‐catalyzed ones; 16.89 (DPPH radical scavenging activity), 37.26 (ABTS), 0.59 (FRAP), and 1.85 (CUPRAC) mmol TEAC/g for pepsin (0.75 g)/Alcalase (0.25 g)‐catalyzed ones; 15.23 (DPPH radical scavenging activity), 22.77 (ABTS), 0.36 (FRAP), and 0.97 (CUPRAC) mmol TEAC/g for pepsin‐catalyzed ones. This distinction in the results can be related to the degree of hydrolysis produced by different enzymes and/or peptides with specific amino acid compositions. For instance, bioactive peptides produced by using Alcalase demonstrated superior antioxidant activity. Presumably, a higher degree of hydrolysis led to lower molecular weights and, consequently, higher antioxidant activity (Rezvankhah et al. 2021a). In a study investigating the antioxidant activity of flaxseed proteins that were hydrolyzed using Alcalase, pepsin, trypsin, and pancreatin, it was observed that the maximum DPPH radical scavenging effect was demonstrated by Alcalase, followed by pancreatin, pepsin, and trypsin, respectively. Concerning the results of ABTS, the maximum values were observed in the samples hydrolyzed by Alcalase and pancreatin, whereas the lowest value was noted for the sample hydrolyzed by pepsin. The authors emphasized that these findings depended on the degree of hydrolysis and the enzyme type. Furthermore, the parameters of the enzymatic process, specifically the acidic or alkaline pH in accordance with the enzyme type, may also influence this phenomenon (Akbarbaglu et al. 2019). Wang et al. (2021) examined the antioxidant activity of cottonseed protein hydrolysate. Elevated values were observed in the ABTS analysis results of the Alcalase‐treated hydrolysate (Wang et al. 2021). A different study conducted enzymatic hydrolysis of cottonseed protein using Alcalase, Flavourzyme, and neutrase. The antioxidant activities were evaluated in hydrolysates generated by various enzymes. Hydrolysates treated with Alcalase displayed strong antioxidant activity in DPPH radical scavenging activity and iron (III) ion‐reducing power (FRAP) tests. The fluctuations in the reducing activity of the hydrolysates might be attributed to the differences in the side chain groups of amino acids. As hydrolysis advances, these groups facilitate the release of electron‐rich sites, thereby liberating peptides and free amino acids. This process provides additional electron and proton sources, contributing to the protection of a high redox potential (de Oliveira Filho et al. 2021). The potential antioxidant activity of lupin protein hydrolysates, which were hydrolyzed using papain, was examined employing four distinct methods: DPPH radical scavenging activity, ABTS, FRAP, and CUPRAC. Maximum antioxidant activity values for hydrolysates compared to the other three techniques were detected with the ABTS technique (Garmidolova et al. 2022). Ultimately, the findings regarding the DH and biological activity of pomegranate protein‐derived peptides manifested that the feasible conditions for enzymatic hydrolysis were a 1:20 (w/w) Alcalase/protein ratio. Thereafter, the bioactive peptides were prepared under the mentioned conditions and stored at −20°C for further applications.

### 3.2 Amino Acid Composition

**FIGURE 2:**
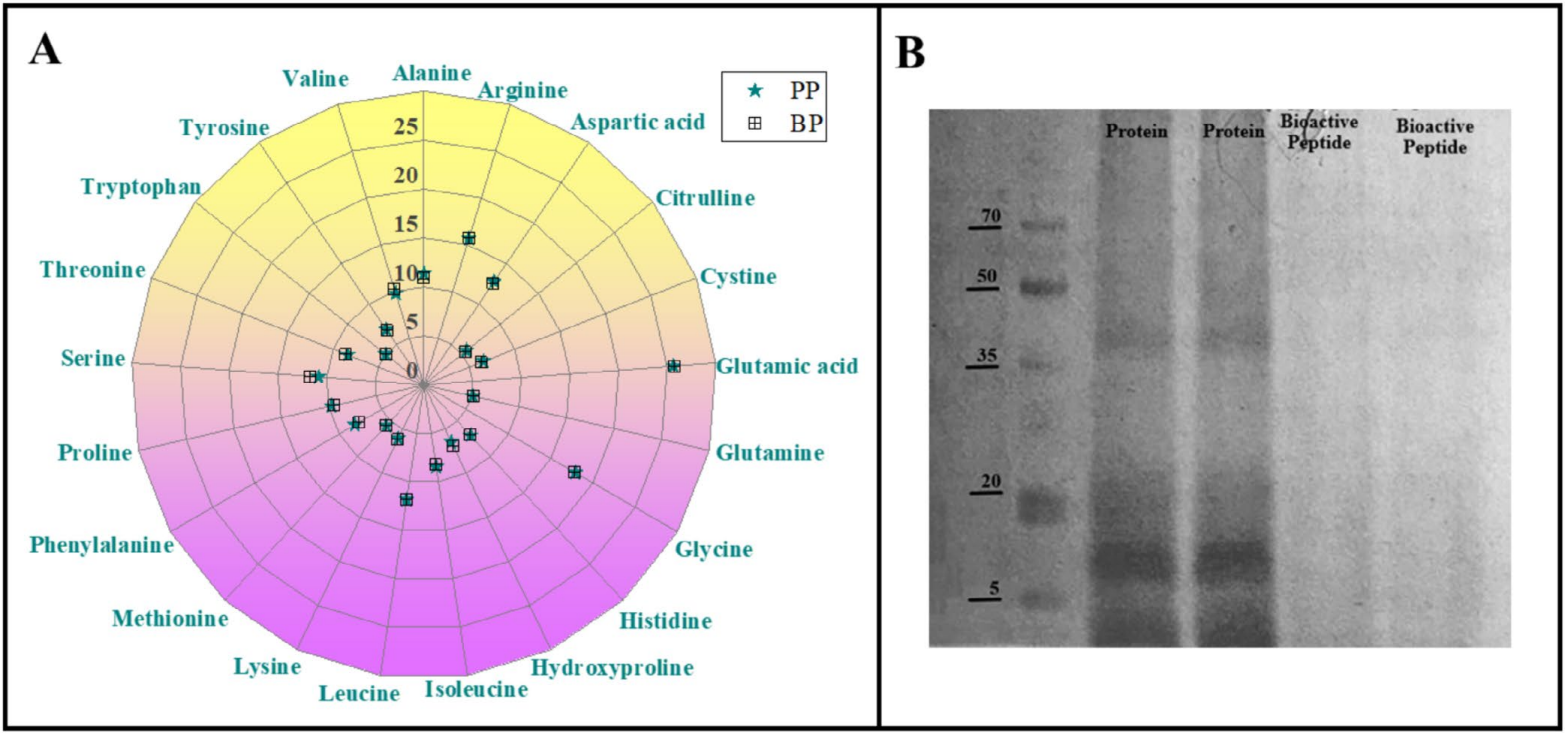
Amino acidic composition (A) and SDS‐PAGE profile (B) of pomegranate seed protein and bioactive peptides. BP, bioactive peptide; PP, pomegranate seed protein.

The amino acid composition of PP and BP was explored in this section, and the related findings are illustrated in Figure 2A. Essential amino acid values for PP and BP were 36.13% and 36.15%, in that order. Resembling essential amino acids, the content of non‐essential amino acids in both of them (PP: 63.87% and BP: 63.85%) was close to each other. Converting *Mucuna pruriens* proteins into peptides leads to slight changes in the levels of essential and non‐essential amino acids of raw material (Herrera Chalé et al. 2014). In another study, hydrolysates were generated from lentil protein using Alcalase and Flavourzyme, and their amino acid composition was analyzed. Consistent with our findings, the amino acid profile of the hydrolysates did not exhibit notable alterations compared to the protein. This situation suggests that enzymatic hydrolysis can preserve the amino acid profile of proteins (Rezvankhah et al. 2021b). As for their individual forms, the predominant essential amino acids in PP and BP were arginine, leucine, and valine. Plant proteins are rich in arginine (Latif et al. 2013). Among non‐essential amino acids, glutamic acid in PP and BP was ahead, followed by glycine, aspartic acid, alanine, and serine. High glutamic acid content is characteristic of plant proteins and their derivatives (Sharma et al. 2014; Yücetepe et al. 2021). Bioactive peptides were generated from green lentil protein using Alcalase and Flavourzyme in a study conducted by Rezvankhah et al. (2021a) and Rezvankhah et al. (2021b). Among the dominant essential amino acids were arginine, leucine, and valine, while glutamic acid, glycine, aspartic acid, and alanine were identified as the predominant non‐essential amino acids (Rezvankhah et al. 2021a). Presumably, the presence of these amino acids in BPs contributed to biological activity. Other studies have documented the contributions of arginine, histidine, aspartic acid, glycine, and glutamic acid in enhancing antioxidant activity. Furthermore, glycine, leucine, and alanine augment the antidiabetic efficacy of protein hydrolysates and function as prospective therapeutic agents for the treatment of hypertension and diabetes (Abbasi et al. 2022). These approaches aligned with the outputs (antioxidant and antidiabetic activity) in the current study.

### 3.3 SDS‐PAGE

The molecular weight of protein and Alcalase‐produced peptides was profiled by SDS‐PAGE, and the related images are presented in Figure 2B. Electrophoretic protein bands with a molecular weight below 50 kDa were observed in PP. Four distinct bands at 46, 44, 32, and 2 kDa were identified in the SDS‐PAGE pattern of protein. The findings regarding SDS‐PAGE profile proteins belonging to the pomegranate seed were in line with the UniProt database (Guzmán‐Lorite et al. 2022a). Also, a similar molecular weight distribution for pomegranate seed‐derived materials was noted elsewhere (Guzmán‐Lorite et al. 2022b, 2022a). Even the authors stated that these characteristic bands correspond to bifunctional fatty acid conjugate/delta (12) oleate desaturase (46 kDa), delta (12) acyl lipid desaturase (44 kDa), acidic endochitinase Pun g 14, amyloplastic (32 kDa), amyloplastic, and pommaclein/punein (2 kDa) in the structure. As for the situation after the enzymatic hydrolysis process, Alcalase provoked the hydrolysis of PP, and all of the characteristic bands disappeared. Bands representing proteins are not seen in the photographs related to the SDS‐PAGE profile when they are exposed to enzymatic hydrolysis (Zhang et al. 2023). In a study conducted by Chang et al. (2021), soy, wheat, and pea proteins were subjected to enzymatic hydrolysis, and their SDS‐PAGE profiles were analyzed. The findings revealed that none of the three plant‐based protein samples exhibited protein bands after hydrolysis. The researchers attributed this phenomenon to the enzymes' hydrolysis ability (Chang et al. 2021). Similarly, in another study, bioactive peptides were produced from wheat gluten, and their molecular weight distribution was examined. The results indicated that bioactive peptides did not have protein bands corresponding to higher molecular weights (≥ 15 kDa). This situation was interpreted as evidence of effective protein degradation or the cleavage of peptide bonds in the proteins (Sotoudeh and Azizi 2024). In soy protein hydrolysates hydrolyzed with fungal protease, the enzyme also caused degradation of the main protein fractions, leading to the disappearance of the main bands seen for the protein (Martínez et al. 2007). Conversion of proteins into structures with smaller molecular weights is responsible for the absence of bands (Rahimipanah et al. 2022). Also, the event regarding the absence of bands in the SDS‐PAGE profile of proteins subjected to enzyme processing verified the bioactive peptide production.

### 3.4 FTIR Spectra

**FIGURE 3:**
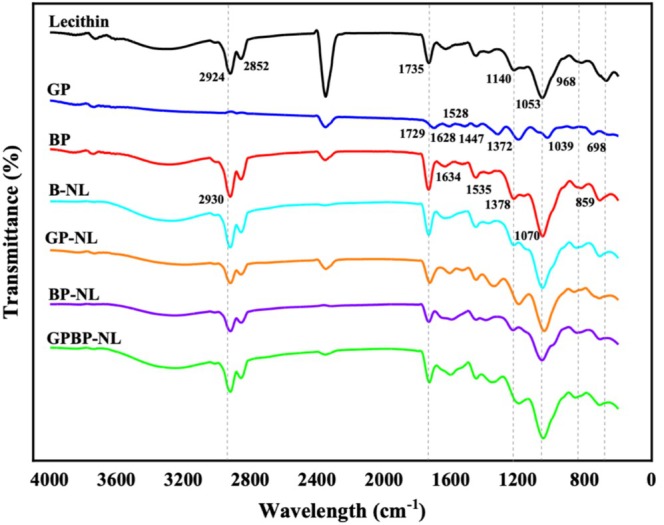
FTIR spectra of nanoliposomes. B‐NL, Nanoliposome prepared without phyto‐active compounds; BP, Bioactive peptide; BP‐NL, Peptide‐loaded nanoliposome; GP, Gall phenolic powder; GPBP‐NL, Both phenolic‐ and peptide‐loaded nanoliposome; GP‐NL, Phenolic‐loaded nanoliposome.

The FTIR technique was employed to elucidate interactions between materials (lecithin, GP, and BP) constituting the nanoliposome system. Therefore, infrared absorption/transmission of all raw materials and nanoliposomal systems was investigated via FTIR spectroscopy, and the related spectra are depicted in Figure 3. A broad peak of lecithin, GP, and BP in the region of 3200–3600 cm^−1^ was related to the hydroxyl (–OH) groups corresponding to alcoholic esters, phenols, *etc*. The peaks regarding the lecithin molecule in wavelengths of 2924 cm^−1^ and 2852 cm^−1^ were assigned to alkane group stretching (CH_2_). Absorbency originating from phospholipid in this molecule appeared at 1735 cm^−1^ representing C=O stretch (between hydrophobic and hydrophilic groups of lecithin). Other molecular fingerprints of lecithin were seen at 1140 cm^−1^ (PO_2_‐ vibration), 1053 cm^−1^ (P–O–C vibration), and 968 cm^−1^ (PO_2_ and P–O–C vibrations) (Vergara and Shene 2019). With respect to GP, the existing peak at 1729 cm^−1^ demonstrated the existence of carboxylic acid. Other wavenumbers regarding C–O stretching, NH bending of amines, C–H bending of alkenes, C–NO_2_ groups of nitro components, C–O stretching, and C–H bending of alkenes in GP were around 1628, 1528, 1447, 1372, 1039, and 698 cm^−1^, in that order (Abdullah et al. 2019). An absorption band for BP was observed in the wavelength of 2930 cm^−1^, correlating with O–H stretch. Amide groups, which are the fingerprint for protein‐derived structures, appeared in the region of approximately 1634 cm^−1^ (Amide I), 1535 cm^−1^ (Amide II), and 1378 cm^−1^ (Amide III). These three peaks corresponded to C–O bending (1634 cm^−1^), C–N bending/NH groups (1535 cm^−1^), and C–N/N–H stretch (1378 cm^−1^). The last characteristic peaks were observed at 1070 cm^−1^ (C–O bending) and 859 cm^−1^ (N–H bending) in BP spectrums (Sarabandi and Jafari 2020). Regarding curves of nanoliposomal systems, all peaks representing the raw materials were clearly seen in the FTIR spectra of these systems, indicating that the loading process of phenolics and peptides into nanoliposomes was performed in a desirable manner. Furthermore, a slight red shift (a lower wavenumber) and a blue shift (a higher wavenumber) in the peaks were observed; however, these shifts were negligible. This observation suggests that physical interactions, rather than chemical events, predominated in nanoliposome systems. In vitamin‐loaded (Pezeshky et al. 2016), polyphenol‐loaded (Lu et al. 2011), and essential oil‐loaded (Sanei‐Dehkordi et al. 2022) nanoliposomes, processes are completed without chemical reactions. Additionally, the study involving the encapsulation of yarrow phenolic compounds in nanoliposomal systems indicated that no chemical reaction occurred, no new peaks were formed, and the existing peaks remained unchanged (Razghandi et al. 2024). Similarly, in the study in which oleocanthal, oleacein, oleuropein, and hydroxytyrosol polyphenols were loaded into ultraflexible nanoliposomes coated with sodium cholate, it was reported that no major shifts occurred in the spectral peaks and no new peaks were formed (Li et al. 2025). On the other hand, in a study where p‐coumaric acid was encapsulated in nanoliposomes, the formation of hydrogen bonds and hydrophobic interactions indicated that this active ingredient was effectively embedded within the phospholipid bilayer (Liu et al. 2024).

### 3.5 Morphology of Nanoliposomes

**FIGURE 4:**
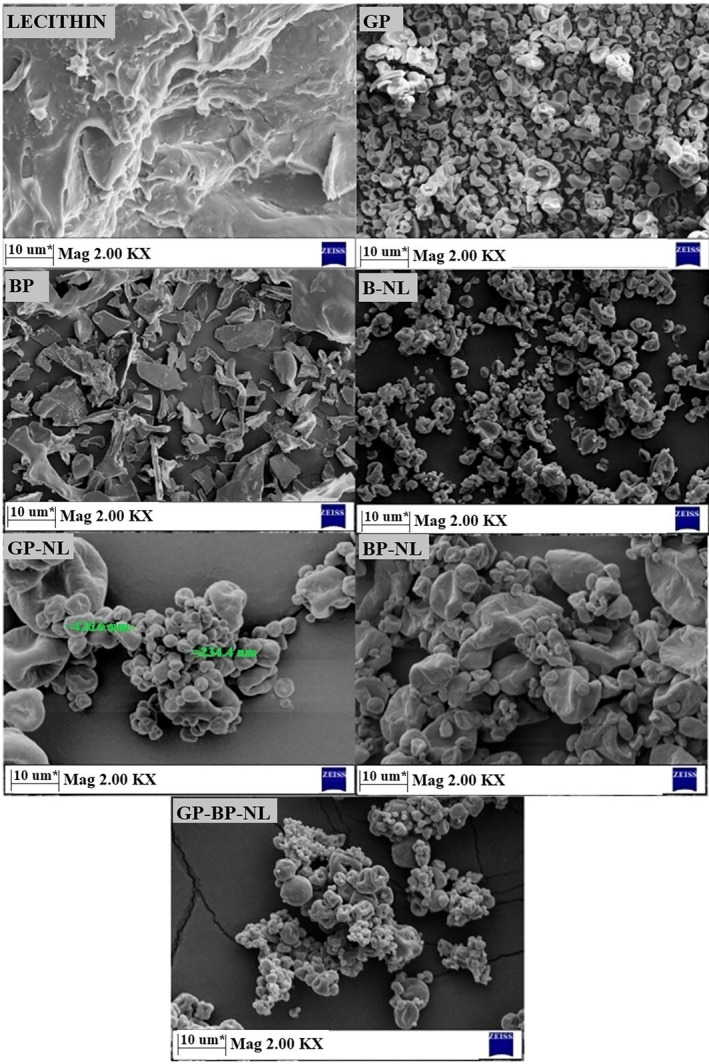
SEM images of nanoliposomes. B‐NL, nanoliposome prepared without phyto‐active compounds; BP, bioactive peptide; BP‐NL, peptide‐loaded nanoliposome; GP, all phenolic powder; GPBP‐NL, both phenolic‐ and peptide‐loaded nanoliposome; GP‐NL, phenolic‐loaded nanoliposome.

This chapter focused on morphological structures of nanoliposome systems, and their photographs are presented in Figure 4. The lecithin morphology exhibited a complex sheet‐like structure. Additionally, irregular layered structures were noticeable in the morphological structure of the purified lecithin powder. The presence of shrinkage and pits on the surface of GPs was prominent. Furthermore, spherical and irregular microcapsules without cracks or holes in the structure were present. On the other hand, visible broken lamellar structures appeared in BPs. In other words, structures of various sizes and shapes, fragmented into numerous folds, were evident. Similar structures were also observed in peptides derived from the enzymatic hydrolysis of pomegranate seed protein (Rahimipanah et al. 2022). Densely accumulated clusters of polydisperse spherical nanoparticles with smooth surfaces and irregular structures were observed in nanoliposomes prepared without phyto‐active compounds (B‐NL). Ellipsoidal particles for nanoliposomes were reported elsewhere (Zhang et al. 2022). Notable variations in the original morphology of the blank system were not identified when GP and BP were loaded into nanoliposomes. In other words, resembling B‐NL, phenolic‐loaded nanoliposome (GP‐NL), peptide‐loaded nanoliposome (BP‐NL), and both phenolic and peptide‐loaded nanoliposome (GPBP‐NL) displayed a consistent spherical configuration. The presence of phyto‐active compounds within liposomal systems has no serious impact on their original morphological structures (Luo et al. 2022). Regarding the advantages of sphericity, presumably, GP‐NL, BP‐NL, and GPBP‐NL possess longer distribution pathways and thicker lipid layers than those of non‐globular nanoparticles. This characteristic feature makes them stand out in pharmaceutical and nutraceutical applications (Sarabandi, Jafari, et al. 2019; Sarabandi, Mahoonak, et al. 2019). On the other hand, partial agglomerate (fusion) shapes in active compound‐loaded nanostructures were obvious. That is, loading GP and BP caused changes in the assembly structure of liposomes, resulting in the increment of their particle size. These findings were supported by particle size values of B‐NL, GP‐NL, BP‐NL, and GPBP‐NL. These values in nanoparticles containing active structures were greater compared to those of their counterpart without GP and BP when particle size results were checked. All these events regarding morphological structures were congruent with those of nanoparticles containing phenolics from pistachio green hull extract (Rafiee et al. 2017) and flaxseed protein hydrolysates (Sarabandi, Jafari, et al. 2019; Sarabandi, Mahoonak, et al. 2019). Additionally, B‐NL, GP‐NL, BP‐NL, and GPBP‐NL were devoid of cracks and/or disruptions. Porous structures in all systems were not seen. The absence of these formations prevents the permeability of gases into the nanoliposome systems and the easy exit of the active substance from these systems. In other words, the absence of deformation in the structure was evidence that the active ingredients are better preserved. To put it more simply, SEM images showed that nanoliposomes are reasonable candidates for preserving phenolics and peptides. Also, these explanations and approaches were consistent with high encapsulation efficiency (86.23%–90.30%). A positive correlation between high encapsulation efficiency and the absence of fissures in capsule systems was reported in previous studies (Molina Ortiz et al. 2009; Yücetepe et al. 2021).

### 3.6 Characteristic Properties of Nanoliposomes

**FIGURE 5:**
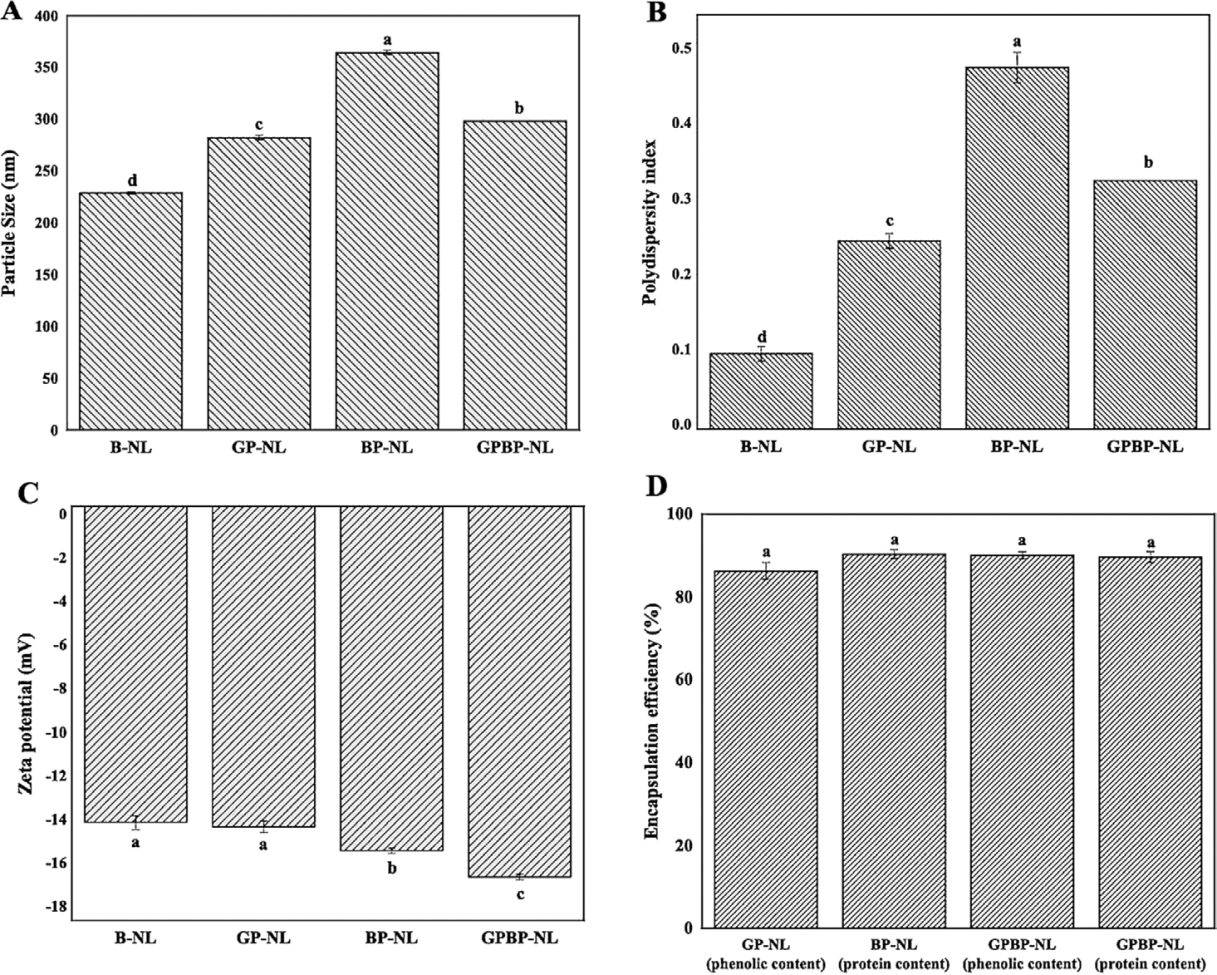
Particle size (A), polydispersity index (B), zeta potential (C), and encapsulation efficiency (D) of nanoliposomes. Results are given as mean ± standard deviation (*n* = 3). Different lowercase letters indicate the differences between samples (*p* < 0.05). B‐NL, anoliposome prepared without phyto‐active compounds; BP‐NL, peptide‐loaded nanoliposome; GPBP‐NL, Both phenolic‐ and peptide‐loaded nanoliposome; GP‐NL, phenolic‐loaded nanoliposome.

The characteristic features of nanoliposomes, namely particle size, PDI, zeta potential, and encapsulation efficiency, were elaborated in this section, and the related findings are depicted in Figure 5. Adding bioactive substances into nanoliposome systems led to remarkable changes in these parameters, except for encapsulation efficiency (*p* < 0.05). Also, the type of active material loaded into these systems affected the results regarding particle size, PDI, and zeta potential. Particle sizes were 228.90, 282.30, 364.30, and 298.30 nm for B‐NL, GP‐NL, BP‐NL, and GPBP‐NL, respectively (Figure 5A). The increase in particle size with the loading of active substances could be ascribed to the replacement of voids within the liposome cores by phenolics and peptides, leading to flexibility and expansion of the liposome (Chotphruethipong et al. 2020). Similar findings were noted in quercetin‐loaded nanoliposomes; specifically, the incorporation of quercetin led to the development of larger structures (Melchior et al. 2023). In a separate investigation, limonene was integrated into liposomal systems at varying concentrations, and it was observed that structures with larger particle sizes were produced in comparison to unoccupied liposomes (Huang, Fang, et al. 2024). Resembling the particle size findings, the minimal values in terms of PDI were detected in B‐NL (0.10), followed by GP‐NL (0.25), GPBP‐NL (0.33), and BP‐NL (0.48) (Figure 5B). This means that all systems exhibited a relatively uniform and narrow particle size distribution (note: the threshold value for PDI is 0.5). Nanoliposomes with PDI > 0.5 are not uniform (Seyedabadi et al. 2021). A study reported PDI values for PEGylated liposomal doxorubicin (PLD, Caelyx) loaded with a leptin‐derived peptide (Lp31) in four different concentrations: 25, 50, 100, and 200 ligands. The PDI values for Caelyx, 25Lp31‐Caelyx, 50Lp31‐Caelyx, 100Lp31Caelyx, and 200Lp31‐Caelyx were 0.069, 0.089, 0.128, 0.179, and 0.191, respectively (Shahraki et al. 2021). In the study examining the loading of miltefosine into liposomal systems, the PDI value increased from 0.240 (blank) to 0.272 (loaded with miltefosine) (Alharthi et al. 2024). As for electrophoretic mobility, the zeta potential values for all nanoliposomes were negative (B‐NL: −14.50 mV, GP‐NL: −14.70 mV, BP‐NL: −15.80 mV, GPBP‐NL: −17:00 mV) (Figure 5C). Electrophoretic mobility results of liposomes are below zero because of the existence of phosphate groups in phospholipids and vary between nearly zero and −61 mV (Luo et al. 2020; Ng et al. 2018; Soema et al. 2015). In the research pertaining to liposomes modified with fatty acids of varying chain lengths (decanoic and stearic acid), peptides were incorporated into these systems, and the zeta potential of peptide‐loaded stearic acid liposomes (−22.87 mV) was found to be higher than that of the blank liposomes (−18.7 mV) (Huang, Song, et al. 2024). Encapsulation efficiency of the nanoliposome systems was 86.23% (GP‐NL), 90.30% (BP‐NL), 90.02% (TPC within GPBP‐NL) and 89.56% (BP within GPBP‐NL) (Figure 5D). In other words, more than 85% of the phyto‐active compounds were loaded into liposomes. This value is higher than 60%, which is the threshold value for high encapsulation efficiency, indicating that an effective encapsulation process was conducted (Ekrami et al. 2023). In a study where casein hydrolysates were incorporated into nanoliposomes, the encapsulation efficiency was reported to range from 77.35% to 92.95%, depending on the type of hydrolysate (Sarabandi, Jafari, et al. 2019; Sarabandi, Mahoonak, et al. 2019). Spirulina LEB‐18 phenolic extracts were loaded into liposomes formed using rice (S‐RL) and soybean (S‐SL) lecithin, and the encapsulation efficiency values were 97.35% and 88.28% for S‐RL and S‐SL, in that order (Machado et al. 2019) (note: beneficial action of high encapsulation efficiency on bioaccessibility was detailed in the in vitro digestion section).

### 3.7 In Vitro Gastrointestinal Tract

Up to today, numerous datasets on biological activities (antioxidant capacity, antimicrobial activity, enzyme inhibition behavior, and so on) of phyto‐active substances have been noted in the scientific literature. The studies regarding the antioxidant capacity of pomegranate peel (Wanderley et al. 2023), the antimicrobial activity of nanoemulsified *eucalypt* extracts (Koshovyi et al. 2023), the inhibition behavior against α‐amylase of *Onosma pulchra* (Sarikurkcu et al. 2020), and ACE of powdered peppermint/spearmint extracts (Cam et al. 2020) could be given as examples of these applications. When these previous studies are examined, biological activities were analyzed directly in the relevant extracts and/or their powder (encapsulated) forms via spectroscopic methods. However, the same materials do not exhibit similar behavior in in vivo conditions. The underlying reason for this is that they are broken down by digestive enzymes, resulting in the reduction of bioavailability. Therefore, examining the behavior of relevant biological materials in the simulated human digestive tract is one of the reasonable ways to provide a more effective roadmap for future studies. A previous dataset emphasized that specific polyphenols could be effectively absorbed through the oral and gastric mucosa, thereby underscoring the significance of modeling each digestive phase (Pineda‐Vadillo et al. 2016). For example, the bioaccessibility of bioactive structures in propolis from the oral to the intestinal stage was examined elsewhere (Mutlu et al. 2024). In this context, this section detailed the behaviors (bioaccessibility and biological activities) of GP and BP loaded into nanoliposomes in the in vitro gastrointestinal tract (from oral to intestinal phase).

### 3.8 Bioaccessibility

**FIGURE 6:**
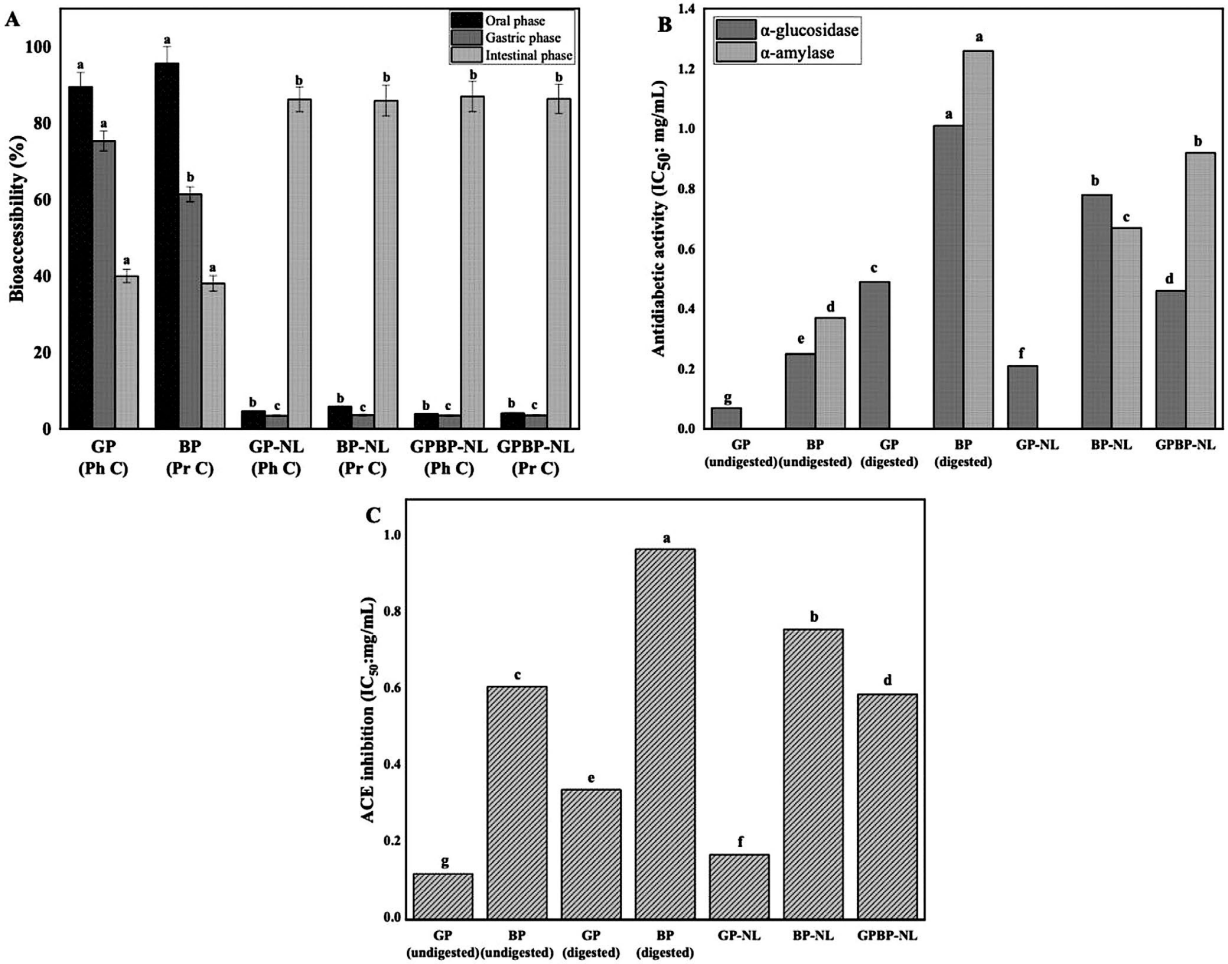
Bioaccesibility (A), antidiabetic activity (B), and ACE inhibition behavior (C) of nano‐encased gall phenolic powders and bioactive peptides. Results are given as mean ± standard deviation (*n* = 3). Different lowercase letters indicate the differences between samples (*p* < 0.05). B‐NL, nanoliposome prepared without phyto‐active compounds; BP, bioactive peptide; BP‐NL, peptide‐loaded nanoliposome; GP, gall phenolic powder; GPBP‐NL, both phenolic‐ and peptide‐loaded nanoliposome; GP‐NL, phenolic‐loaded nanoliposome; Ph C, phenolic content; Pr C, protein content.

GP, BP, GP‐NL, BP‐NL, and GPBP‐NL were exposed consecutively to simulated oral‐to‐colon stage for detecting bioaccessibility of phenolics and peptides. In other words, the potential of nanoliposome systems as delivery systems was investigated, and the related findings are depicted in Figure 6A. The bioaccessibility for GP, BP, GP‐NL, BP‐NL, GPBP‐NL phenolics, and GPBP‐NL peptides in the oral phase was 89.54%, 95.7%, 4.67%, 5.83%, 3.96%, and 4.12%, in that order. A significant decline in this value of GP (75.37%) and BP (61.42%) was noted during the gastric digestion process. An early study demonstrated a notable reduction in free anthocyanin levels from the oral to the gastric phase, indicating that gastric conditions greatly affect the availability of bioactive compounds (Teixeira et al. 2024). The bioaccessibility values for each nanoliposome type were comparable to those observed during gastric digestion (GP‐NL: 3.45%, BP‐NL: 3.64%, GPBP‐NL: 3.51% for phenolics, GPBP‐NL: 3.58% for peptides). Digestion occurs in a short time (2 min) in the oral phase, which is devoid of enzymes that affect lipid‐based carrier systems. Furthermore, liquid nanoliposomes do not require chewing, allowing them to pass into the gastric phase in a much shorter time. In this context, most studies on the in vitro digestibility of liposomal systems have not included the oral phase in the digestion (Ismail et al. 2021). Similarly, liposomal structural integrity remains virtually unchanged under gastric conditions. This is because phospholipids cannot be hydrolyzed in the stomach, as gastric lipase has no activity on phospholipids (Deng et al. 2024). Furthermore, liposomal membranes have a well‐organized structure, which ensures their structural stability against gastric environmental stress (Weilin Liu et al. 2019). This means that the integrity of the stable liposome is not altered, and the liposomal membrane prevents the substances it entraps from leaking into the stomach (Liu et al. 2020). Therefore, liposomes are not severely damaged in these two phases of the digestive tract, meaning that a large portion of their phenolics and peptides reaches the intestine. The positive action of these systems on the bioaccessibility of biological structures in the intestinal phase was obvious (*p* < 0.05). Bioaccessibility values for free (unencapsulated) forms of phenolics and peptides were 40.05% and 38.09%, in an order. That is, more than 50% of these compounds were degraded after the simulated digestion. The proportion of unencapsulated polyphenols reaching into the blood circulatory system is approximately 30% of their amounts initially exposed to digestion (Toro‐Uribe et al. 2018). The bioaccessibility percentage for spirulina LEB‐18 phenolics at the end of the colonic digest was 31.65 elsewhere (Machado et al. 2019). This value was identified as 51.35% in unprotected osteogenic peptide (Zhu, Cheng, and Du 2024; Zhu, Ma, et al. 2024). The result for the native form of egg white peptide in the micellar phase was 57% in another study (Du et al. 2020). Ultimately, when phenolics and peptides without any protection were subjected to harsh environments of in vitro simulated digestion, their stability was not well maintained. Similar behavior for bioactive materials was noted elsewhere (Faridi Esfanjani et al. 2018). Until reaching the intestinal phase and in intestinal conditions, direct contact of biological materials in native forms with enzymes (*i.e*., α‐amylase, pepsin, and trypsin)/low pH environments that have versatile destructive impacts on them could be the explanation for this phenomenon (Mor et al. 2021). As for the findings regarding the bioaccessibility of nanoliposome‐encased GP and BP, notable outputs for the scientific literature emerged. Sharp increments in the bioaccessibility level of these functional groups were seen when the nanoliposome process was applied. In fact, more than 2‐fold distinctions in digestive stability were defined compared to uncoated GP and BP (*p* < 0.05). One of the plausible strategies for enhancing bioaccessibility of phyto‐active structures is to load them into nanoliposome systems (Nascimento et al. 2023). Over 85% GP and BP bioaccessibility in all nanoliposomes (GP‐NL: 86.25%, BP‐NL: 85.92%, GPBP‐NL: 87.03% for phenolics, GPBP‐NL: 86.41% for peptides) was achieved. Bioaccessibility values for catechin, epicatechin, and ferulic acid when encapsulated within nanoliposomal systems shifted from 40%–50% to 70% and 85% compared to their native forms (Kasapoğlu et al. 2024). Similarly, side/undesirable effects of mouth, stomach, and intestinal mediums on peptides were hindered via nanosized systems, and their level of inclusion into the bloodstream by encasing with these systems was increased from 57% to 84% (Du et al. 2020). The encapsulation of osteogenic peptides within nanoliposomes significantly enhanced their bioaccessibility, increasing from 51.35% to 66.53% (Zhu, Cheng, and Du 2024; Zhu, Ma, et al. 2024). Another study reported that the bioaccessibility of kiwi leaf proanthocyanidins encapsulated in nanoliposomes increased by 2.28–3.07‐fold during in vitro digestion (Lv et al. 2023). Most of the approximately 15% loss in GP and BP happened in the final stage of digestion (intestinal phase). The underlying reason for these losses might be explained by two approaches. The first of these is the low resistance of liposomes to the intestinal conditions because of the presence of lipase causing their hydrolysis. The existence of bile salts in the related fluid, which forms a strong bond with hydrophobic structures such as phospholipids, is the second of these approaches. The bile salts could penetrate the interior of nanoliposomal systems, resulting in swelling and disintegration of the relevant systems. In both cases, liposomes are damaged, and the transition of nano‐encased active structures from the system to intestinal fluid occurs (Raftani Amiri et al. 2024). Similar descriptions regarding the behavior of nanoliposomal systems in the gastrointestinal tract were reported in previous studies (Basyigit 2023; Hu et al. 2023). Also, the beneficial action of high encapsulation efficiency on bioaccessibility was identified in previous datasets (Basiri et al. 2017). Regarding the details of this relationship, if the majority of functional structures are on the surface rather than the interior of the nanoliposomes, these structures could be easily released into the relevant medium in the digestive tract, and their bioaccessibility decreases. These comments were supported by the results found in the current study. The encapsulation efficiency and bioaccessibility values for phenolics and peptides loaded in nanoliposomes were around 90% and 85%, in that order.

### 3.9 Antidiabetic Activity

The main function of digestive enzymes, namely α‐glucosidase and α‐amylase, is to convert polysaccharides into their monomers in the human body. In other words, these enzymes play a role in the hydrolysis of carbohydrate‐derived macrostructures into their monomers (monosaccharides) that have the potential to enter the blood circulatory system. Uncontrollable inclusion of these monomers into the bloodstream induces hyperglycemia and type 2 diabetes (Yu et al. 2024). Thus, controlling the actions of α‐glucosidase and α‐amylase in the body is essential in terms of health. Nowadays, the most widespread way used in hindering the activity of these enzymes is oral intake of synthetic drugs, such as acarbose. However, the long‐term/excessive usage of acarbose disrupts the body's balance. For example, previous findings showed that it triggers weight loss in the body (Golalipour et al. 2024). For this reason, numerous studies have been conducted on natural counterparts of this drug, and scrutinies on this subject persist. Among the promising candidates, polyphenols are ahead (de Paulo Farias et al. 2021). Moreover, in the last couple of years, the role of peptides in the inhibition of α‐glucosidase and α‐amylase has aroused curiosity, and the incidence/prevalence of datasets regarding this topic has increased in the scientific literature recently (Islam et al. 2022; Prakash Nirmal et al. 2023). In this part of the study, all aspects of GP and BP, which are promising natural alternatives in treating diabetes, were investigated. The inhibition level of GP and BP against α‐glucosidase and α‐amylase before and after digestion was examined. Also, the antidiabetic potential of nanoliposome‐encased GP and BP (B‐NL, GP‐NL, BP‐NL, and GPBP‐NL) in the micelle phase was inspected. All findings are depicted in Figure 6B. The action of α‐glucosidase was violently suppressed by undigested GP (IC_50_ value: 0.07 mg/mL). The reason behind this inhibition is the non‐covalent bonds formed between phenolics and α‐glucosidase (Martinez‐Gonzalez et al. 2017). However, GP had no aptitude to impede α‐amylase activity, meaning the phenolics‐enzyme interaction was blocked by starch (Aleixandre et al. 2022). Resembling these findings, earlier investigations noted the beneficial action of gall extracts toward the inhibition of α‐glucosidase (IC_50_ value: 0.02 mg/mL) but not α‐amylase (Başyiğit et al. 2020). The datasets regarding the eminent impact of *Lamiaceae* plant‐derived phenolics on the active center of α‐glucosidase were recorded elsewhere. Conversely, the authors reported that the same situation was not the case for amylase (Kwon et al. 2006). Unlike GP, undigested BP was involved in modulating the action of both digestive enzymes (IC_50_ value: 0.25 mg/mL for α‐glucosidase and 0.37 mg/mL for α‐amylase). Peptides obtained from albumin (Yu et al. 2012) and black cricket protein (de Matos et al. 2022) hinder α‐glucosidase and α‐amylase actions. The potential of peptides from *Ginkgo biloba* seed protein as an α‐glucosidase inhibitor with an IC_50_ value of 12.94–17.82 mg/mL was approved (Wang et al. 2023). In a study, peptide production from camel whey proteins was performed under various conditions (selected independent factors: temperature, enzyme concentration, and process time) and the IC_50_ value for α‐amylase was in the range of 0.29–3.69 mg/mL (Baba et al. 2021). Various mechanisms underlie the inhibitory behavior of peptides toward α‐glucosidase and α‐amylase. The existence of hydrophobic amino acids, including alanine, valine, leucine, isoleucine, phenylalanine, and methionine in BP may be responsible for α‐glucosidase inhibition. Presumably, thanks to these amino acids, the peptides linked to the active site of the enzyme through hydrophobic interactions and ultimately displayed their inhibitory impact. A relationship between hydrophobic amino acids and α‐glucosidase inhibitory activity of bioactive peptides is evident (Ren et al. 2016). In the case of α‐amylase inhibition, an interaction between aromatic amino acid residues in the catalytic site of the enzyme and the related substrate is involved. In all likelihood, the aromatic amino acids in the peptide interacted with these residues, resulting in the management of α‐amylase/glucose homeostasis. Briefly, α‐amylase inhibition might be ascribed to aromatic‐aromatic interactions between the peptide and the enzyme (Vilcacundo et al. 2017). As for the digestion step, when bioactive compounds, including polyphenols and peptides, are subjected to gastrointestinal digestion, their enzyme inhibition abilities are affected either positively or negatively (Gutiérrez‐Grijalva et al. 2019; Mudgil et al. 2021). In the current study, digested GP (IC_50_ value: 0.25 mg/mL for α‐glucosidase) and BP (IC_50_ value: 1.01 mg/mL for α‐glucosidase and 1.26 mg/mL for α‐amylase) displayed lower enzyme inhibitory capacity than those of undigested counterparts. An explanation for this phenomenon could be the partial degradation of phenolics and peptides until reaching the circulation. This approach was supported by the bioaccessibility findings of nanoliposome‐encased GP and BP. When checking these findings, the degradation rate was lower in encapsulated GP and BP compared to uncoated ones. In parallel with bioaccessibility results, GP and BP loaded into nanoliposomes exhibited superior antidiabetic activity in the micellar phase juxtaposed with their digested native forms. A study reported that loading goat milk whey protein peptides into liposomes significantly improved the inhibitory activities of α‐glucosidase and α‐amylase enzymes after simulated gastrointestinal digestion (Du et al. 2022). The high levels of antidiabetic activity were ascribed by the authors to the enzymatic hydrolysis process, which facilitates the release of hydrophobic amino acids. In addition, peptides with a lower molecular weight demonstrate stronger hypoglycemic activity (Ren et al. 2016). In another study, flavonoids extracted from fenugreek seeds loaded into chitosan‐coated nanoliposomes exhibited significant inhibitory activities against α‐amylase and α‐glucosidase (Ashraf et al. 2025). The antidiabetic mechanism was explained by hydrogen bonding, interactions related to aromatic compounds, the influence of electrostatics, and dipole–dipole interactions (Śliwińska‐Hill and Wiglusz 2020). In addition, the interaction between selenoproteins and lipid metabolism has been the subject of current research in order to investigate its antidiabetic effect (Liang et al. 2024). The IC_50_ value for GP‐NL, BP‐NL, and GPBP‐NL as α‐glucosidase inhibitors was 0.21, 0.78, and 0.46 mg/mL, in that order. Also, this value for α‐amylase was detected in BP‐NL (0.67 mg/mL) and GPBP‐NL (0.92 mg/mL) but not in GP‐NL. Ultimately, the potential of GP and BP in treating hyperglycemia and type 2 diabetes emerged. Their undigested native forms showed supreme results in inhibiting α‐glucosidase and α‐amylase. The presence of hydrophobic amino acids in pomegranate seed protein BP and aromatic compounds of GP was associated with the antidiabetic effect. However, as emphasized in different parts of the text, the behavior of bioactive compounds in the gastrointestinal tract provides guidance on whether they should be involved in any application (especially in the treatment of diseases). Ultimately, when phyto‐active structures passed through the digestive tract without any protective shield, a decrease in their activity against enzymes was observed. On the other hand, when they were trapped in nanoliposome systems, more promising findings for future applications were obtained.

### 3.10 ACE Inhibition

The renin–angiotensin–aldosterone system (RAAS) is accountable for managing blood pressure in the human body. To prevent blood flow from decreasing, renin fabricated in the kidneys breaks down angiotensinogen and produces the inactive molecule angiotensin‐I (Ang‐I). ACE converts Ang‐I to angiotensin‐II (Ang‐II), a vigorous vasoconstrictor. Ang‐II binds to cell surfaces, causing a series of reactions and increasing blood pressure (Festa et al. 2020). In other words, the uncontrolled presence of ACE in the bloodstream causes the pressure in the blood vessels to rise above the desired values (hypertension) (Wenceslau et al. 2021; Yoshie‐Stark et al. 2004). Hypertension is controllable by inactivating ACE in various ways, namely synthetic drugs (Kokubo and Matsumoto 2016). However, synthetic drugs cause side effects, namely chronic dry cough and angioedema (de Castro and Sato 2015; Li et al. 2015). Therefore, natural ACE inhibitors, like phenolic compounds and/or plant‐based peptides, are becoming increasingly substantial. There have been many studies investigating ACE inhibitors of bioactive compounds (Cam et al. 2020) and plant‐based peptides (Daskaya‐Dikmen et al. 2017). In this section, the ACE inhibition effect of digested/undigested natural inhibitor GP and BP, as well as GP and BP entrapped in nanoliposomes (after digestion), was evaluated. ACE inhibition values of all samples are given as IC_50_. An inferior IC_50_ value demonstrates effective inhibition (Pedroche et al. 2002). The results of ACE inhibition activity are given in Figure 6C. The IC_50_ value of the undigested GP was defined as 0.12 mg/mL. GP's powerful ACE inhibition effect is associated with its phenolic acids (Elham et al. 2021). Hydrogen bonds could have been developed between the zinc and varied regions of the protein in ACE by the hydroxyl (‐OH) ions of phenolic acids (Margalef et al. 2017). Sharifi et al. (2013) investigated the ACE inhibition effect of gall plant barks and reported an inhibition effect (approximately 94%) at a concentration of 0.33 mg/mL (Sharifi et al. 2013). For another plant material (*Phaleria macrocarpa*), ACE inhibitor activity was between 0.12 and 0.16 mg/mL (Radji et al. 2013). As for undigested BP, the ACE inhibition test was found to have an IC5_0_ value of 0.61 mg/mL. This phenomenon was attributed to amino acids including proline, arginine, and lysine in BP (Guzmán‐Lorite et al. 2022a; Vásquez‐Villanueva et al. 2015). Moreover, acidic amino acids, namely aspartic acid and glutamic acid, are known to own hypocholesterolemic efficacy (Erdmann et al. 2008). ACE inhibition activity for wheat gluten bioactive peptides (IC_50_ value: 0.68 mg/mL) (Liu, Zhang, et al. 2021) and grass carp peptide hydrolysates (IC_50_ value: 0.69 mg/mL) (Chen et al. 2016) were investigated elsewhere.

ACE inhibition effects of phenolic compounds and bioactive peptides continue to be investigated. As far as is known, most studies are conducted on the biological activity of plant extracts. That is, tests are performed directly on the extracts without any digestion process. However, to know the bioavailability of the extracted functional components, their digested value must also be evaluated. For this reason, in vitro digestion of GP and BP was performed, and their ACE inhibition effects were appraised (Figure 6C). The ACE inhibition ability of GP was reduced and the IC_50_ value shifted from 0.12 to 0.34 mg/mL. The reduction in ACE inhibition activity of *Berberis* plants was approximately 10% after digestion. The authors reported that this reduction occurred since phenolics did not pass through the membrane into the serum fraction (Şensu et al. 2021). Resembling GP, ACE inhibiting property of the digested BP was diminished. BP exhibited an IC_50_ value of 0.97 mg/mL after in vitro digestion. Peptides could be broken down by the catalytic effects of enzymes in the gastrointestinal system, and eventually their inhibitory effect might be reduced (Sharma et al. 2022). Briefly, GP and BP lost some of their bioavailability due to digestive enzymes, and the ACE inhibitory behavior decreased. Therefore, the ACE inhibition effects of GP and/or BP endowed in nanoliposomes were re‐evaluated after in vitro digestion. The inhibitor impact of nano‐encased phyto‐active structures was superior in the micellar phase compared to their unencapsulated forms, and IC_50_ values for GP‐NL, BP‐NL, and GPBP‐NL were 0.17, 0.76, and 0.59 mg/mL, in that order. This trend was in line with bioaccessibility results. Another finding regarding these results was that ACE inhibition behavior of GPBP‐NL was between the GP‐NL and BP‐NL; meaning GP and BP did not exhibit antagonistic effects on each other (Awika and Duodu 2017). Different studies have reported incompatible datasets for the effects of nanoliposome systems on the ACE inhibition activities of bioactive substances. For example, in a study on sheep whey hydrolysates, a decrease in ACE activity was observed after they were loaded into nanoliposome systems (Corrêa et al. 2019). On the other hand, following simulated gastrointestinal digestion, encapsulated stonefish biopeptides were shown to have improved inhibitory effects against ACE compared to unencapsulated ones. The study highlighted that peptides with shorter sequences have a strong capacity to act as ACE inhibitors (Auwal et al. 2018). Microcapsules and nanocapsules lead to the increased ACE inhibitory activity of phenolic extracts from jujube peel in the in vitro digestion environment. Despite exposure to different pH levels and enzyme interactions, the coated phenolics exhibited strong stability and continued to show ACE inhibitory activity post in vitro gastrointestinal digestion (Şensu et al. 2024). In other studies, ACE inhibition activity of nano‐encased biological structures was close to those of their unencapsulated forms at constant concentration (Hanachi et al. 2022; Mecheta et al. 2020; Mosquera et al. 2016). Considering all the findings, undigested GP and BP showed strong ACE inhibitory behavior. The notable ACE inhibitory properties observed in this investigation were ascribed to the enhanced hydrogen bonds (–OH) found in GP, as well as the abundance of hydrophobic (alanine, valine, leucine, isoleucine, phenylalanine, and methionine), acidic (aspartic acid and glutamic acid), and positively charged (lysine and arginine) amino acids in BP. However, for bioactive ingredients to exert their bioactivity, they must be stable in the digestive tract and enter the blood circulatory system. Direct exposure of GP and BP to gastrointestinal digestion resulted in reduced bioactivity. On the other hand, their effect on the enzyme was enhanced by inclusion in nanoliposome systems.

## 4 Conclusions

The current study presented datasets related to bifunctional systems. High bioaccessibility of phyto‐active structures is one of the prerequisites for formulating innovative healthy foods using them and incorporating them into pharmacological applications. Another requirement for their usage in the specified applications is that they are as stable as possible during gastrointestinal digestion and exhibit high biological activity in the micellar phase. The native forms of both bioactive structures, namely BP and GP, exhibited low bioaccessibility (approximately 40%). On the other hand, the bioaccessibility of GP‐NL, BP‐NL, and GPBP‐NL was close and exceeded 80%. Based on these findings, utilizing nano‐encased active ingredients (phenolics and peptides) rather than their native forms in food and pharmacological applications is more reasonable. Notable enhancements in antidiabetic and ACE inhibitory activities were observed exclusively in the presence of GP alone within NL. However, a comparable level of development for these biological activities was not attained with BP alone. In other words, the results indicated that the combined use of BP and GP, instead of using peptide alone, was a more rational approach to achieving improved biological activity within the gastrointestinal tract. In future studies, utilizing nanocarriers, particularly GP‐NL or GPBP‐NL, for the development of functional foods is a potentially impactful idea. Also, these functionalized nanocarriers could be incorporated into edible films to impart biological activity. However, supporting these further studies with scale‐up applications is essential for the transition to industry. Due to their high bioaccessibility, GP‐NL, BP‐NL, and GPBP‐NL are also promising tools for the management of medical conditions. On the other hand, this approach needs to be supported by rat studies and clinical applications. Additionally, in future studies, multiple structures with different functional properties might be loaded into a single nanoliposome system to investigate their usage potential in the treatment of certain diseases.

## Author Contributions

**Melike Yücetepe:** conceptualization (equal), data curation (equal), investigation (equal), methodology (equal), software (equal), writing – original draft (equal), writing – review and editing (equal). **Mehmet Şükrü Karakuş:** formal analysis (equal), investigation (equal), methodology (equal), resources (equal), writing – original draft (equal). **Merve Akalan:** formal analysis (equal), investigation (equal), software (equal), writing – original draft (equal). **Kamile Bayrak Akay:** formal analysis (equal), investigation (equal), software (equal), writing – original draft (equal). **Hidayet Sağlam:** formal analysis (equal), methodology (equal), visualization (equal), writing – original draft (equal). **Asliye Karaaslan:** formal analysis (equal), methodology (equal), visualization (equal), writing – original draft (equal). **Bülent Başyiğit:** conceptualization (equal), data curation (equal), investigation (equal), resources (equal), writing – original draft (equal), writing – review and editing (equal). **Mehmet Karaaslan:** conceptualization (equal), data curation (equal), funding acquisition (equal), investigation (equal), resources (equal), supervision (equal), writing – original draft (equal), writing – review and editing (equal).

## Conflicts of Interest

The authors declare no conflicts of interest.

## Data Availability

The data that support the findings of this study are available on request from the corresponding author. The data are not publicly available due to privacy or ethical restrictions.
